# Social and physical environmental correlates of movement behaviors, body weight status, and well-being among adolescents in Nigeria: a youth-centered participatory action project narrative review

**DOI:** 10.1186/s12966-026-01904-1

**Published:** 2026-03-17

**Authors:** Adewale L. Oyeyemi, Adefunke B. Ogunleye, Anita C. Okafor, Neha Kollipara, Mai J.M. ChinAPaw, Dayo R. Omotoso

**Affiliations:** 1 College of Health Solutions, Arizona State University, Phoenix, AZ 85004 USA; 2 Department of Physiotherapy, Redeemer’s University, Ede, Nigeria; 3 Gender and Education Department, Value Re-orientation for Community Enhancement (VARCE), Osogbo, Nigeria; 4 Barrett Honors College, Arizona State University, Tempe, USA; 5 Public and Occupational Health, Amsterdam UMC Location VUmc, Amsterdam, The Netherlands; 6 Health Behaviors and Chronic Diseases and Methodology, Amsterdam Public Health Research Institute, Amsterdam, The Netherlands; 7 Department of Human Anatomy, Redeemer’s University, Ede, Nigeria

**Keywords:** Physical activity, Sedentary behavior, Overweight, Obesity, Health, Teenager, Africa

## Abstract

**Background:**

The epidemic of non-communicable diseases related to lifestyle movement behaviors is rising rapidly in Africa, with adolescents increasingly affected by physical inactivity, excessive sedentary behavior, poor sleep, being overweight or obese, as well as mental health challenges. While evidence supports the importance of social and physical environmental factors in improving adolescents’ health, understanding these issues among African adolescents remains limited. Insights from evidence from low- and middle-income countries (LMICs), which are often underrepresented in research on environmental influences on movement behaviors and health, may be relevant to certain Western contexts, especially those in resource-constrained settings. This narrative review aims to synthesize findings from available literature on social and physical environmental correlates of movement behaviors, body weight status, and well-being among adolescents in Nigeria, an LMIC in Africa.

**Methods:**

We searched electronic databases, including PubMed, Scopus, Google Scholar, and African Journals Online, for original research articles published prior to January 2025 on socioenvironmental factors and movement behaviors, body weight status, and well-being among Nigerian adolescents (aged 10–20 years). Each study underwent screening and data extraction by two reviewers. Study quality was assessed with the modified Downs and Black checklist, and data were analyzed using narrative synthesis.

**Results:**

A total of 25 articles met the inclusion criteria, all of which were cross-sectional studies. The review suggested that social class, school settings, peer relationships, family structure, electronic device use in the bedroom, neighborhood walkability features, availability of open recreational spaces, and urbanization were important socioenvironmental factors related to multiple movement behaviors and health outcomes among Nigerian adolescents. Some of these relationships were modified by gender and socioeconomic status.

**Conclusions:**

The evidence from this narrative review highlighted the need for more prospective and intervention studies to understand how environmental factors may promote healthier movement behaviors, enhance health, and overall well-being among Nigerian adolescents. The results could be used by researchers, policymakers, and stakeholders, including school administrators, urban planners, health professionals, parents, and community organizations who are interested in how social and physical environments contribute to adolescents’ movement behaviors, health, and well-being in resource-constrained settings.

**Supplementary Information:**

The online version contains supplementary material available at 10.1186/s12966-026-01904-1.

## Background

Non-communicable diseases (NCDs), including cardiovascular diseases, cancers, chronic respiratory diseases, diabetes, and obesity, are rapidly increasing across Africa [1, 2]. NCDs currently account for approximately 37% of deaths in sub-Saharan Africa, an increase of 24% since 2000 [3], and are projected to become the leading cause of mortality by 2030, surpassing deaths due to communicable diseases in the region’s countries [4]. In Nigeria, NCDs are already responsible for about 29% of all deaths [5], and projected to continue to rise if urgent actions are not taken [6]. However, NCDs originate at a younger age, are of long duration, and result from a combination of factors, including genetics and physiological, environmental, and lifestyle behaviors [7].

Adhering to the guidelines for lifestyle movement behaviors, such as physical activity, sedentary behavior, and sleep, has been associated with better health outcomes among adolescents, including reduced obesity, improved psychosocial well-being, and protection against NCDs [8–10]. Despite these health benefits, about 81% of adolescents worldwide do not meet the physical activity guidelines, placing them at a greater risk of developing NCDs [11]. Moreover, only 10% of adolescents internationally are achieving the 24-hour movement guidelines for physical activity, sedentary time, and sleep, suggesting the importance of promoting these behaviors from early ages [12]. Thus, interventions are needed not only to increase the proportion of young people who meet physical activity recommendations but also to adhere to guidelines on screen time and sleep duration. Also, improving other health outcomes, such as body weight status and well-being, among adolescents is critical as it brings benefits today, for decades to come, and for the next generation [13]. However, interventions and strategies to promote lifestyle movement behaviors and health among adolescents must be evidence-based and context-specific.

Environmental and policy interventions are generally recommended for promoting lifestyle movement behaviors and health because they can influence large groups and bring about population-wide changes [14, 15]. Ecological models of health behaviors, which emphasize the importance of multiple levels of influence (individual, interpersonal, organizational, environmental, and policy) in explaining health behaviors and obesity [16], are the most commonly used theoretical frameworks for understanding such interventions. In this context, multiple review studies have linked aspects of the urban built environments (e.g., neighborhood walkability, green space, access to parks, recreation facilities, pedestrian and cyclist facilities, aesthetics, traffic safety, and safety from crime) to physical activity and body weight status among adolescents [17–21]. However, most of this evidence was from studies conducted in Western high-income countries [20, 21], and did not examine other important lifestyle movement behaviors, such as sedentary behaviors or sleep, or health outcomes such as psychosocial well-being [22, 23]. Given that the built environment, transportation infrastructure, and urban development patterns differ in developing African countries compared to those in Western high-income countries, review studies need to synthesize African-specific evidence to inform evidence-based interventions in the region.

A recent systematic review of six African-based cross-sectional studies on the associations between built environments and physical activity found that only two (i.e., crime and traffic safety) of seven environmental constructs were associated with physical activity among African children and adolescents [24]. However, this study did not synthesize evidence on the link between environmental factors and sedentary behavior and sleep, nor related health outcomes, such as obesity or poor psychosocial well-being, that are major health issues among adolescents in Africa [25, 26]. Beyond the physical, built environments, social environmental factors (e.g., supportive family structure, peer relationships, family socioeconomic status [SES] or social class, school setting, neighborhood social capital, and community safety and engagement) are important for adolescents’ health and well-being [27, 28]. Thus, more review studies on how social and physical environments are related to movement behaviors, body weight status, and psychosocial well-being among young people in African countries could help inform effective planning that is appropriate and relevant to the local context. This is especially critical for Nigeria, where the population is still very young (median age of 18.1 years) [29] and the epidemic of lifestyle movement behavior-related NCDs is rising rapidly [5, 6].

With an estimated population of approximately 36.4 million youth (projected to double to 79.4 million by 2050), Nigeria has the largest population of youth aged 10–24 years on the entire African continent [30]. Thus, examining evidence on how social and physical environmental factors are related to lifestyle movement behaviors and health outcomes among adolescents in Nigeria is an important public health priority. This could support and reinforce locally relevant structural solutions and contribute to international evidence-based socioenvironmental initiatives aimed at improving movement behaviors and reducing NCD risks in adolescents globally. As nations having most of the world’s population are underrepresented in health publications [31, 32], and populations and their health needs are constantly evolving [33], insights and evidence from low- and middle-income countries (LMICs) are urgently needed. Moreover, such evidence may also be applicable to certain Western contexts, especially to resource-constrained settings and marginalized communities (e.g., immigrant groups, indigenous populations, low-income neighborhoods) [33, 34]. Therefore, the present narrative review aims to summarize the social and physical environmental correlates of physical activity, sedentary behavior, sleep, body weight status, and well-being among adolescents in Nigeria, an LMIC. This study is part of the Youth-centered Participatory Action (YoPA) project, which aims to optimally tailor, implement, and evaluate social and physical environmental interventions; YoPA uses an evidence-informed co-creation approach for structural improvement in the lifestyle of adolescents in urban vulnerable life situations in two European and two African cities [35].

## Methods

One of the goals of the YoPA project is to summarize existing literature and combine the resulting evidence with local needs and priorities to inform the development of systems maps of factors that support and reinforce adolescents’ movement behaviors and well-being [35]. Since this is the first comprehensive summary of evidence on physical and social environmental factors of movement behaviors and health among adolescents conducted in Africa, we employed a narrative review approach to synthesize existing primary studies conducted among Nigerian adolescents. This method provides a flexible, rigorous, and comprehensive overview of the current state of knowledge, highlights areas for further study, develops new perspectives, and informs practice [36, 37]. To address the underrepresentation of African adolescents’ research in Western-dominated academic literature and databases, we applied a search strategy that incorporated diverse sources. Specifically, we searched multiple databases, including Google Scholar, PubMed, Scopus, and African Journal Online, for research articles published before January 1, 2025, on the association of physical and social environmental factors with movement behaviors, body weight status, and well-being among Nigerian adolescents younger than 20 years.

We used the following search terms to standardize and streamline our search strategy across the different databases: “Built environment” OR “Neighborhood environment” OR “Urban environment” OR “Community environment” OR “Social environment” OR “Urban” OR “Social factors” AND “Physical activity” OR “Exercise” OR “Sport” OR “Sedentary time” OR “sitting” OR “Sedentariness” OR “Screen time” OR “TV viewing” OR “Phone use” OR “Computer use” OR “ Sleep” OR “Sleep disorders” OR “Sleep quality” OR “Wakefulness” OR “Overweight” OR “Obesity” OR “Body mass index” OR “Well-being” OR “Quality of life” OR “Mental health” OR “Mental disorders” AND “Adolescents” OR “Youth” OR “Teenagers” AND “Nigeria”. Additionally, we reviewed reports from intergovernmental organizations, such as the World Health Organization and Nigerian-based non-governmental organizations (e.g., the Nigerian Heart Foundation), for relevant citations and references of primary studies.

To be included in the review, studies had to be original research reports on quantitative or qualitative analyses of the association of social and physical environmental factors with one or more movement behaviors and/or body weight status and/or well-being among Nigerian adolescents. The mean age of participants had to be between 10 and 20 years for studies to qualify. Only original articles published in English before January 1, 2025, were considered eligible; therefore, reviews, conference abstracts, commentaries, and editorials were deemed ineligible.

We used a modified version of the Downs and Black checklist to evaluate the methodological quality of the included studies [38]. Since all included studies employed a cross-sectional design, items relevant to intervention studies (e.g., randomization process, losses to follow-up, etc.) were excluded from the modified checklist. The modified scale consisted of ten items related to the quality of reporting (6 items), external validity (2 items), and internal validity/risk of bias (2 items). Each item was scored as “Yes” (1) or “No”/ “Unable to determine” (0), and the total item scores were calculated as a percentage of “Yes” ratings per study and across all included studies. Two reviewers (ABO and NK: 13 papers; ACO and DRO: 12 papers) independently assessed the included studies using the modified checklist, and any disagreements were resolved through discussion or with a third reviewer (ALO) if consensus could not be reached.

### Data extraction and analysis

Standardized data extraction tables were created to retrieve information related to the study design (year, region/state of study, number of participants, age, gender), methodology, main outcome and exposure variables, measurement tools, and key study findings. For quality control purposes, five reviewers (ALO, ABO, ACO, DRO, and NK) extracted the data, and a second reviewer (ALO or NK) cross-checked the screened records and data entries for accuracy. Reviewers were not blinded to the authors or journals when extracting data. Data were analyzed using narrative synthesis and presented in thematic narratives and tables. We grouped the themes based on our main outcomes (i.e., physical activity, sedentary behaviors, sleep, body weight status, and well-being) and associated social and physical environmental factors.

## Results

### Search results

Table 1 gives an overview of the main characteristics of the included articles. A total of 25 articles focusing on the social and physical environmental correlates of physical activity (8%), sedentary time (16%), sleep (28%), body weight status (28%), and well-being (20%) among Nigerian adolescents (aged 10–20 years or mean age < 20 years) met the selection criteria. One article focused on both physical activity and sedentary activities [39]. Most of the studies (44.0%) were published between 2010 and 2019, with 40% originating in the most recent 5-year period (2020–2024). All included studies employed a cross-sectional survey design; 96% utilized a quantitative method, and one (4%) adopted a mixed method. The sample size of the included studies ranged from 49 to 1799 participants. When organized by region, the majority of the studies (51.9%) were conducted in the Southwest of Nigeria, followed by the Southeast (14.8%) and Northcentral (14.8%), with four studies each.

**Table 1:** Study Characteristics (*n* = 25)

Characteristics	Categories	*N*	%
Year of publication	2000–2009	4	16.0
	2010–2019	11	44.0
	2020–2024 (Dec)	10	40.0
Region (Nigeria)*	South East	4	14.8
	South South	1	3.7
	South West	14	51.9
	North Central	4	14.8
	North East	2	7.4
	North West	2	7.4
Sample size	< 100	2	8.0
	100–499	12	48.0
	500–999	4	16.0
	1000–above	7	28.0
Study design	Cross-sectional	25	100.0
	Longitudinal	0	0.0
	Experimental intervention	0	0.0
Study method	Quantitative	24	96.0
	Qualitative	0	0.0
	Mixed method	1	4.0
Study focus**	Physical activity	2	8.0
	Sedentary behavior	4	16.0
	Sleep	7	28.0
	Body weight status	7	28.0
	Well-being	5	20.0

* One of the studies (Ene-Obong et al. [40]) was conducted across three regions (South East, South South, and South West) of Nigeria. **One study focused (Ajayi [39]), on both physical activity and sedentary behavior

### Methodological quality

Table 2 shows the results of the methodological quality assessment of the 25 included studies. Overall, 12 (48.0%) of the studies had excellent quality scores of 100%, while the remaining studies had slightly lower quality scores, ranging from at least 90% (*n* = 7, 28.0%) to 80% (*n* = 6, 24.0%). Lower scores were due to either the targeted and recruited participants not being representative of the population or no estimates of random variability being reported.

**Table 2:** Quality appraisal of included studies

Author [citation]	Q1	Q2	Q3	Q4	Q5	Q6	Q7	Q8	Q9	Q10	Total %
Ajayi [39]	Y	Y	Y	Y	Y	Y	Y	Y	Y	Y	100.0
Oyeyemi et al. [41]	Y	Y	Y	Y	Y	Y	Y	Y	Y	Y	100.0
Odusoga & Sholeye [42]	Y	Y	Y	Y	Y	Y	Y	Y	Y	Y	100.0
Otinwa & Ademola [43]	Y	Y	Y	Y	Y	Y	N	N	Y	Y	80.0
Ezezue et al. [44]	Y	Y	Y	Y	Y	Y	N	N	Y	Y	80.0
Anjana et al. [45]	Y	Y	Y	Y	Y	Y	Y	Y	Y	Y	100.0
Peter et al. [46]	Y	Y	Y	Y	Y	Y	Y	N	Y	Y	90.0
Olorunmoteni et al. [47]	Y	Y	Y	Y	Y	Y	Y	Y	Y	Y	100.0
Sanya et al. [48]	Y	Y	Y	Y	Y	Y	Y	Y	Y	Y	100.0
Omotosho et al. [49]	Y	Y	Y	Y	Y	Y	Y	Y	Y	Y	100.0
Balogun et al. [50]	Y	Y	Y	Y	Y	Y	Y	Y	Y	Y	100.0
Olorunmoteni et al. [51]	Y	Y	Y	Y	Y	Y	Y	Y	Y	Y	100.0
Maduabuchi et al. [52]	Y	Y	Y	Y	Y	Y	Y	N	Y	Y	90.0
Ejike et al. [53]	Y	Y	Y	Y	Y	Y	Y	N	Y	Y	90.0
Olumakaiye [54]	Y	Y	Y	Y	Y	Y	Y	Y	Y	Y	100.0
Omigbodun et al. [55]	Y	Y	Y	Y	Y	Y	Y	Y	Y	Y	100.0
Ben-Bassey et al. [56]	Y	Y	Y	Y	Y	Y	N	Y	Y	Y	90.0
Adegoke et al. [57]	Y	Y	Y	Y	N	Y	Y	N	Y	Y	80.0
Ojofeitimi et al. [58]	Y	Y	Y	Y	N	Y	Y	N	Y	Y	80.0
Ene-Obong et al. [40]	Y	Y	Y	Y	Y	Y	Y	Y	Y	Y	100.0
Modupe [59]	Y	Y	Y	Y	Y	Y	N	N	Y	Y	80.0
Cheng et al. [60]	Y	Y	Y	Y	Y	Y	N	N	Y	Y	80.0
Nnubia & Emmanuel [61]	Y	Y	Y	Y	Y	Y	Y	N	Y	Y	90.0
Alhassan et al. [62]	Y	Y	Y	Y	Y	Y	Y	N	Y	Y	90.0
Asukuti et al. [63]	Y	Y	Y	Y	Y	Y	Y	N	Y	Y	90.0
Total (%) Yes	100	100	100	100	92	100	80	48	100	100	

Yes (Y), No (N), and Unclear (U). Quality appraisal question Q1: Was the objective clearly stated? Q2: Were the main outcomes clearly described? Q3: Were participants’ characteristics (inclusion and exclusion criteria) clearly defined? Q4: Were the main findings of the study clearly described? Q5: Were estimates of random variability in the data reported? Q6: Were actual probability values reported? Q7: Was the sample targeted representative of the population? Q8: Were the subjects who participated in the study representative of the population? Q9: Was appropriate statistical analysis used (e.g., non-parametric methods used for small sample size)? Q10: Were the primary outcome measures used accurate (valid and reliable, e.g., outcome measure clearly described)?

### Physical activity

Table 3 and Supplementary Table 1 present the review results for socioenvironmental factors in relation to adolescents’ physical activity. A study exploring open space typologies (i.e., school playground, neighborhood park, incidental open spaces, and pocket park) utilization for outdoor activities in residential neighborhoods found school playgrounds (63.3%) and incidental open spaces (27.3%) as the most common space typologies used for physical activity among adolescents in Nigeria [39]. The study revealed significant variation in self-reported vigorous outdoor activities across the open space typologies, with most vigorous activities occurring in incidental open spaces (49.6%), followed by school playgrounds (40.1%) and pocket parks (29.4%). The least amount of vigorous physical activities occurred in the neighborhood park (23.2%). Additionally, there was a notable difference in activity levels between males and females in the city, with more males utilizing the open spaces due to cultural and social expectations [39]. Similarly, another study found that walkability features of the neighborhood environments, such as access to destinations (β = 0.18; CI = 0.67, 2.24), residential density (β = 0.10; CI = 0.01, 1.74), and the availability of pedestrian infrastructure (β = 0.14; CI = 0.49, 2.68), were associated with increased leisure-time physical activity and active transportation to school among Nigerian male adolescents [41].

**Table 3:** Study design and summary of findings on environments and outcome variables of interest

Study	Design	Region	Sample size	Age	Outcome variables	Summary of findings on environmental factors and outcome variables*
Ajayi, 2021 [39]	Cross-sectional (mixed method)	South West	1265	14–19 years	Physical activity and Sedentary behavior	Incidental open space and school playgrounds were commonly utilized for physical activity, while parks were the most utilized open space typologies for sedentary activities.
Oyeyemi et al., 2014 [41]	Cross-sectional	North East	1006	12–19 years	Physical activity	Walkability features of the neighborhood environment were positively related with physical activity.
Odusoga & Sholeye, 2021 [42]	Cross-sectional	South West	330	15–19 years	Sedentary behavior	Home electronic and social environments (using screen-based devices) were positively related with sedentary behavior.
Otinwa & Ademola, 2017 [43]	Cross-sectional	South West	60	10–19 years	Sedentary behavior	Screen time was elevated among urban resident adolescents.
Ezezue et al., 2021 [44]	Cross-sectional	South East	49	15–24 years	Sedentary behavior	Western-style home environment layout design was associated with more sedentary activities compared with traditional home environment layout.
Anjana et al., 2024 [45]	Cross-sectional/multi-country study	North East	268	11–19 years	Sedentary behavior	Home electronic environment (using screen-based devices) and neighborhood environment features (pedestrian infrastructure and safety) were positively associated with sedentary behavior (screen time, transport-related sitting)
Peter et al., 2017 [46]	Cross-sectional	North West	353	10–19 years	Sleep	Home electronic environment features (watching TV or playing video games) were not significantly associated with sleep problems.
Olorunmoteni et al., 2018 [47]	Cross-sectional	South West	346	10–19 years	Sleep	Home electronic environment (not using computer at night), family structure (polygamous family), and lower social class were positively associated with better sleep quality and patterns.
Sanya et al., 2015 [48]	Cross-sectional	North Central	1033	10–19 years	Sleep	Home electronic environments (making phone calls at night, surf the internet at night) were common problems affecting night time sleep duration.
Omotosho et al., 2022 [49]	Cross-sectional	North Central	512	10–19 years	Sleep	Family structure (first born) and school setting (non-hostel living) were positive predictors of poor sleep.
Balogun et al., 2017 [50]	Cross-sectional	South East	450	10–19 years	Sleep	School setting and parents’ social class were not associated with sleep quality.
Olorunmoteni et al., 2023 [51]	Cross-sectional	South West	448	10–19 years	Sleep	School setting (private school) was associated with sleep quality.
Maduabuchi et al., 2014, [52]	Cross-sectional	South East	443	10–19 years	Sleep	Social class was not associated with sleep latency.
Ejike et al., 2008 [53]	Cross-sectional	North Central	1088	10–20 years	Body weight status	BMI was higher among adolescents in urban areas than those in non-urban areas.
Olumakaiye, 2008 [54]	Cross-sectional	South West	401	10–19 years	Body weight status	Overweight and obesity was higher among adolescents in urban areas than in rural areas.
Omigbodun et al., 2010 [55]	Cross-sectional	South West	1799	10–19 years	Body weight status	Urban environment and private school were associated with higher odds of being overweight.
Ben-Bassey et al., 2007 [56]	Cross-sectional	South West	1504	10–19 years	Body weight status	Prevalence of overweight and obesity was higher in urban areas than in rural areas.
Adegoke et al., 2009 [57]	Cross-sectional	South West	720	6–18 years	Body weight status	Higher social class was positively associated with being overweight.
Ojofeitimi et al., 2011 [58]	Cross-sectional	South West	520	10–19 years	Body weight status	Private school setting was associated with higher rates of being overweight and obese than a public school setting.
Ene-Obong et al., 2012 [40]	Cross-sectional	South East, South South, South West	1599	5–18 years	Body weight status	Prevalence of obesity was highest in the most urbanized cities and among those in high social classes.
Modupe, 2021 [59]	Cross-sectional	South West	270	10–20 years	Well-being	School type with positive teacher attitudes and school located in poor neighborhood were positively and negatively related with social well-being, respectively.
Cheng et al., 2014 [60]	Cross-sectional/multi- country study	South West	449	15–17 years	Well-being	Social supports from family and neighborhoods were positively associated with a greater feeling of hope.
Nnubia & Emmanuel, 2023 [61]	Cross-sectional	South East	836	10–18 years	Well-being	Weak family structure, being in a higher grade at school, and low social class were associated with anxiety and depression in boys and girls.
Alhassan et al., 2024 [62]	Cross-sectional	North West	384	High school adolescents	Well-being	Peer pressure was positively associated with poor psychological well-being.
Asukuti et al., 2023 [63]	Cross-sectional	North Central	400	10–20 years	Well-being	Social interaction avoidance and self-isolation were common strategies for dealing with depression symptoms.

* Detailed findings and measures of environments and outcomes are in Supplementary Additional File Tables 1, 2, 3, 4 and 5

### Sedentary behaviors

Table 3 and Supplementary Table 2 present the review results for socioenvironmental factors in relation to sedentary behaviors conceptualized as sitting time, screen time, and screen-based behavior. The majority of Nigerian adolescents engaged in excessive sedentary behavior (86.7%–92%), and screen-based behavior (79%) [39, 42]. Particularly, the use of smartphones or tablets was the largest contributor to sedentary time among Nigerian adolescents [42]. Notably, adolescents’ sedentary behavior was associated with social environmental factors, including family ownership of motorized transport (a proxy for high social status) and smoking [42]. Relatedly, high screen time among adolescents living in urban areas of Lagos was associated with higher sedentary time and lower physical fitness, thereby increasing the risk of NCDs [43]. This study found that urban living adolescents with high screen time had higher systolic blood pressure (128.34 ± 3.45 mmHg vs. 116.89 ± 2.86 mmHg), body mass index (BMI) (21.34 ± 1.29 kg/m2 vs. 19.31 ± 1.74 kg/m2), and waist-hip ratio (0.87 ± 0.13 vs. 0.71 ± 0.09) compared to those with lower screen time [43]. Another study that examined how urban residential layout design was associated with sedentary behavior in youth found that residents spent about 90% of their time at home on a sedentary activity, with those living in buildings with a traditional house layout spending the least time on a sedentary activity compared to those in dwelling units with a Western-style house layout pattern [44]. The study noted that traditional layout design provided spatial flexibility that encouraged transitioning from sedentary to light activity (standing) [44]. Another study showed that the neighborhood park (92.1%), followed by pocket parks (88.2%), school playgrounds (86.1%), and incidental open spaces (85.2%) were the most utilized open space typologies for adolescent sedentary activities in Nigeria [39]. A recent study on associations of parent-reported neighborhood and adolescent-reported home environments with sedentary behavior among adolescents aged 11–19 years from 14 countries found owning a personal social media account and having electronic devices in the bedroom were significantly associated with higher screen time among Nigerian adolescents [45]. Also, neighborhood pedestrian infrastructure and safety were associated with more transport-related sitting time among adolescents in Nigeria [45].

### Sleep

Table 3 and Supplementary Table 3 present results from studies that examined socioenvironmental factors in relation to sleep among adolescents in Nigeria. All the studies indicated that Nigerian adolescents experience insufficient sleep duration at night [46–52]. One study found that the most prevalent sleep-related problem among adolescents was awakenings during the night (34.6%), followed by excessive daytime sleepiness (21%) [46]. This study identified home environments that encourage TV watching and video game playing up to one hour before bedtime as potential factors that could negatively affect the quality and quantity of sleep among Nigerian adolescents [46]. Another study revealed that a majority of Nigerian school-going adolescents (80.9%) used one or more electronic devices, including cell phone (52.3%) at bedtime, and identified lower social class (aOR = 2.706; 95% CI = 1.179–6.212), lack of computer use at bedtime (aOR = 2.126; 95% CI = 1.081–4.179), and living in a polygamous family (aOR = 4.262 (95% CI = 1.825–9.956) as independent positive predictors of sufficient sleep on weekends among Nigerian adolescents [47]. A study by Omotosho et al. identified male gender, being the firstborn child in the family, and living at home instead of in a school hostel as the most important predictors of poor sleep quality among school-going adolescents [49].

Meanwhile, a recent study provides more insightful results on the relationship between sleep quality, school schedules, and the mental health of Nigerian secondary school adolescents [51]. The study found that school closure time and school types (χ2 = 8.047, *P* = 0.045) were significantly associated with sleep quality, with the majority of adolescents with late closure times (84.2%) and those attending private schools (89.9%) reporting poorer sleep quality. However, school type was independently associated with adolescents’ sleep quality, with the odds of experiencing poor sleep quality roughly doubling among adolescents in private schools compared to those in public schools (aOR = 1.97, 95% CI = 1.069–3.627). The study also indicated that poor sleep quality was related to depression and anxiety [51].

### Body weight status

Table 3 and Supplementary Table 4 present the review results for socioenvironmental factors in relation to body weight status among Nigerian adolescents. One study showed that Nigerian adolescents living in urban areas (boys = 20.74 ± 3.27 kg/m², girls = 21.35 ± 3.37 kg/m²) had significantly higher BMIs than those living in non-urban areas (boys = 20.33 ± 3.11 kg/m², girls = 20.60 ± 2.97 kg/m²) [53]. Similarly, three studies found that the prevalence of being overweight or obese was higher among Nigerian adolescents living in urban environments than those living in rural areas [54–56].

Another study conducted in a semi-urban area of Nigeria found that a higher social class was associated with a higher prevalence of overweight among children and adolescents [57]. Also, attending private school (a proxy for high socioeconomic status) was associated with a higher level of being overweight or obese among girls [58]. Furthermore, higher levels of urbanization and parents’ income were associated with being overweight and obese among school-aged children and adolescents in four cities in Southern Nigeria [40]. The study found that the prevalence of overweight and obesity varied significantly according to location and socioeconomic status, being highest in Lagos (33.8%) compared to Nsuka (14.6%), Port Harcourt (10.3%), and Aba (3.5%), and highest among children and adolescents from very high income > N300,000/month families (25.8%) compared to those with low income < 100,000/month (7.1%) [40].

### Well-being

Table 3 and Supplementary Table 5 present the review results for socioenvironmental factors in relation to well-being. A study that examined the association of environmental factors with the well-being of school-going adolescents with delinquent behaviors in Nigeria showed that school location (β = -0.239, *p* < 0.05) and school type (β = -0.170, *p* < 0.05) were significantly associated with social well-being among adolescents [60]. Attending schools located in poor neighborhoods was negatively (*r* =- 0.268, *p* < 0.05) associated with adolescents’ social well-being, while attending schools with positive teacher attitudes and clear, unambiguous rules was positively (*r* = 0.144, *p* < 0.05) associated with social well-being [59].

In another study, stronger perceptions of having a caring female adult in the home and feeling connected to the neighborhoods were positively associated with levels of hope and negatively associated with depression and posttraumatic stress symptoms among Nigerian adolescents [60]. This study recommended that improving social support in families and neighborhoods may alleviate distress and foster hope among Nigerian adolescents living in economically distressed areas [60]. A study that explored gender variations in the prevalence and correlates of depression and anxiety among Nigerian adolescents identified different socioenvironmental factors for boys and girls [61]. Identified socioenvironmental correlates of poor mental well-being symptoms among boys included having mothers or guardians with higher educational qualifications (i.e., tertiary level education), living with a guardian, not having a close emotional relationship with parents or guardians, and coming from a low-income background. For girls, the correlates included higher age (15–18 years), being in a higher grade (i.e., senior secondary school classes), having an unemployed or retired father or guardian, and not having a close relationship with parents or guardians [61]. Similarly, peer pressure was significantly associated with poor psychological well-being among Nigerian secondary school adolescents [62]. The most frequent sociobehavioral coping strategies employed by school-going adolescents in Nigeria to overcome depression included playing games as a form of distraction (42.3%), avoiding talking to people (44.5%), self-criticism (28.8%), and social isolation (24.5%) [63].

## Discussion

This narrative review synthesized findings from 25 cross-sectional studies, providing for the first time an overview of the evidence on the associations between various social and physical environmental factors and movement behaviors, body weight status, or well-being among Nigerian adolescents. Overall, the main findings indicated that several social and physical environmental factors were independently related with Nigerian adolescents’ physical activity, sedentary time, sleep, body weight status, and well-being, suggesting the need to consider socioenvironmental factors when designing effective active living and health promotion initiatives for adolescents [14, 16, 64]. The most frequently studied movement behavior and health outcomes were sleep and body weight status, followed by well-being, with only two studies focusing on physical activity. This highlighted disparities in research on socioenvironmental correlates of physical activity and sedentary behaviors among adolescents in Nigeria. Perhaps, understanding the correlates of long-standing issues of obesity and mental health disorders in the Nigerian population [5, 65], and the emerging field of sleep epidemiology, were of higher priority for Nigerian researchers than physical activity and sedentary behavior.

### Social environment correlates

Social class, family support, peer relationships, and school settings and dynamics were dominant social environment correlates of Nigerian adolescents’ health and well-being. The studies by Cheng et al. [60] and Alhassan et al. [62] demonstrated the crucial role of social support in helping adolescents manage mental health issues, with peer support being particularly important for their psychological well-being. Also, Nigerian adolescents who engaged in delinquent behaviors often found themselves in unstable social situations, which could lead to lower social well-being [59]. While a higher social class was associated with higher overweight and obesity among Nigerian adolescents [40, 55, 57, 58], a lower social class and living in a polygamous family setting (counterintuitively) were unique social characteristics associated with having sufficient sleep among Nigerian adolescents [47]. Perhaps, Nigerian adolescents from lower social classes and polygamous families have less access to electronic devices at home that disrupt their sleep time compared to those from high social classes and monogamous families. In the African settings, polygamy is linked to limited resources and higher poverty rates that could constrain access to electronic devices in these households, especially for women and children. Moreover, attending private schools (a proxy for a high-SES social environment) rather than public schools was linked to negative sleep outcomes [50, 51], poor social well-being [59], and being overweight or obese [51, 58]. However, poor sleep quality has been related to depression and anxiety among Nigerian adolescents, suggesting that improved could benefit mental health in the general Nigerian adolescent population [51].

Furthermore, important gender differences existed in how social environments are experienced and associated with Nigerian adolescents’ health behaviors and well-being. Nigerian male adolescents compared to their female peers reported lower sleep sufficiency and higher susceptibility to feelings of depression and anxiety, which may be tied to the differential impact of peer relationships and family structure and supports between boys and girls [47, 50, 61]. These findings highlighted the need for gender-sensitive strategies that consider the unique psychosocial experiences of boys and girls in Nigeria.

### Physical environmental correlates

The physical attributes of the neighborhood and home environments, including access to non-residential destinations, high residential density, availability of pedestrian infrastructure, access to recreational open spaces, and activity-friendly home layout designs were associated with higher levels of physical activity among Nigerian adolescents [39, 41, 44]. Interestingly, parks, playgrounds, and incidental open spaces were associated with high levels of both physical activity and sedentary behavior among Nigerian adolescents [39]. Perhaps many Nigerian adolescents will default to using outdoor open spaces for leisure-time sedentary activities, especially when no or few appropriate physical activity facilities are available. Equipping open spaces like parks and playgrounds with appropriate amenities has been advocated as an important strategy for promoting active living among Nigerian adolescents [39, 66]. Also, the home environment characteristics that facilitate having electronic devices in the bedroom, access to a computer, owning a social media account, and living at home (vs. living in school hostels) were enablers of higher screen time and poorer sleep among Nigerian adolescents [45, 47, 49]. These findings highlighted that attributes of the neighborhood and home environments seem relevant for improving multiple health behaviors and related outcomes, including sleep, screen time, and obesity among Nigerian adolescents.

Urban residence was a major correlate of higher body weight status among adolescents in the present review. This is consistent with the behavioral epidemiological transition, characterized by a shift away from traditional diets and lifestyles in favor of more “Westernized” diets and lifestyles (e.g., more animal-based food products, processed food high in saturated fats and sugar, and sedentary service-oriented occupations) that are associated with the escalating levels of obesity, physical inactivity, and chronic disease in many developing countries undergoing rapid urbanization [67–69]. Nigerian adolescents who live in urban areas had higher BMIs and were more overweight or obese than their counterparts in rural areas [53–56]. This finding is a cause for concern because of the global trend toward urbanization. Rapid urbanization, access to processed foods high in saturated fats and sugar, the proliferation of screen-based and mechanized-labor-saving devices, and reduced physical activity have been recognized as potential drivers of obesity, inactive lifestyles, and NCDs in African countries [1–4].

Considering the increasing global urbanization [70], activity-friendly urban design could be an effective strategy to mitigate the impacts of urbanization on the rising epidemic of overweight and obesity in Nigeria [71]. Taken together, these findings on how built environmental factors relate to adolescents’ movement behaviors, body weight status, and well-being in Nigeria provide evidence that could inform potential solutions for the growing epidemics of chronic diseases in Africa and other disadvantaged settings of the world. As part of our multi-country YoPA project [35], evidence from the current narrative review has guided the co-creation and development of a local systems map that identifies factors affecting adolescents’ physical activity and well-being in Nigeria. This evidence is being integrated into a toolbox of actions to guide other disadvantaged settings in both high-income and low-and middle-income countries in adopting the YoPA approach to improving the health of adolescents living in vulnerable circumstances [35].

### Strengths and limitations

This narrative review has several strengths, including the inclusion of studies from all geographic and political regions of Nigeria. This provided a comprehensive coverage and an overview of studies on the relationship between social and physical environmental factors and multiple health behaviors and outcomes among adolescents across diverse settings in an understudied region of the world. Second, given the relative underrepresentation of African-based research in major international databases, such as PubMed/Medline and Scopus, we searched several African journals not indexed in these databases through African Journal Online and Google Scholar. Moreover, this is the first review to assess the association of social and physical environmental factors with different movement behaviors, body weight, and well-being among Nigerian adolescents. Further, the methodological quality of the evidence from the 25 included studies was mostly very good to excellent, suggesting the findings of this review could be used as important correlates to explore when designing future public health research and programs to inform socioenvironmental determinants of adolescents’ health in Nigeria.

Yet, this study has some important limitations that should be acknowledged. First, the non-systematic approach to the review precludes rigor in study selection and amplifies the risk of selection bias. We did not account for the total number of articles identified through our search strategies and those excluded after title and abstract screening across all databases. Narrative reviews are often not reproducible due to the influence of the authors and setting on screening, sampling, and analysis [36]. Second, the varying focus of interests and main outcomes across the included studies made direct comparisons challenging. Third, the reliance on self-reported data in most surveys may have led to underreporting of outcomes and exposures due to social desirability bias. Fourth, publication bias may have influenced the findings, as significant results are more likely to be published, potentially causing an imbalance in this review [72]. Also, we did not report non-significant findings from the articles included in our review, complicating the comparisons of inconsistent findings within and across studies. Finally, all included studies were cross-sectional, prohibiting causal conclusions. However, this review makes an important contribution by providing a comprehensive and current understanding of socioenvironmental correlates of movement behaviors and health outcomes among adolescents in Nigeria. The findings highlighted important individual and structural correlates that could inform the design of future public health intervention research to address the growing NCD problems in Nigerian populations [5, 6].

## Conclusions

The evidence from this narrative review shows that living in urban environments, poor access to non-residential destinations and pedestrian infrastructure, lack of open recreational spaces, attending private school, home environments that promote the use of screen-based devices in the bedroom and reduce time for physical activity, negative peer influence, and having high social class parents are important social and physical environmental factors associated with multiple movement behaviors and health outcomes, including physical inactivity, sedentary time, poor sleep, being overweight or obese, and poor well-being among adolescents in Nigeria. These socioenvironmental factors could be targeted when planning future public health research or actions aimed at improving lifestyle movement behaviors, body weight status, or well-being among adolescents in Nigeria and potentially other LMIC or resource-constrained settings.

The evidence could be useful for researchers, school administrators, urban planners, health professionals, parents, and community organizations who are interested in socially activating activity-friendly home, school, and community environments contributing to adolescents’ health. Future qualitative studies, as well as prospective and intervention research, on socioenvironmental factors and adolescent’s health outcomes are needed to better assess the long-term effects and causal relationships to inform sustainable, context-specific policies in Nigeria. By understanding and addressing these broader socioenvironmental factors, stakeholders and policymakers can improve lifestyle movement behaviors, reduce obesity risks, and enhance the overall well-being of Nigerian adolescents.

## Supplementary Information

Supplementary Material 1.

## Data Availability

All data generated and/or analysed during the current study are included in this published article and its supplementary information files.

## Abbreviations

BMI: Body Mass Index
YoPA: Youth-centered Participatory Action Research
NCDs: Non-communicable diseases
LMICs: Low- and middle-income countries
