# Supplementary material for: Social and physical environmental correlates of movement behaviors, body weight status, and well-being among adolescents in Nigeria: a youth-centered participatory action project narrative review
Source: Int J Behav Nutr Phys Act. 2026 Mar 17;23:42. doi: 10.1186/s12966-026-01904-1 (PMC13107659; doi:10.1186/s12966-026-01904-1)
Supplement: Supplementary file 1 — Supplementary Material 1. [file 12966_2026_1904_MOESM1_ESM.docx]

**Supplementary Table 1**. Physical activity and social and physical environment factors

| Author/Year [Reference] | Study design | Region/City | Sample Size | Age | Outcome Variable | Measurement of outcome variable | Physical and social environment factors | Measurement of environment factors | Main findings with respect to environmental factors and outcome variables |
| --- | --- | --- | --- | --- | --- | --- | --- | --- | --- |
| Ajayi 2021 [39] | Cross-sectional (mixed method) | Southwest/ Osogbo | 1265 | 14-19 years | Physical activity | Structured Questionnaire Survey of physical activity types (vigorous, moderate, walking) | Urban Open Spaces | Direct Observation; Satellite  imagery and  geographic information collected from Google Earth | Playgrounds (63.3%) and incidental open spaces (27.3%) are commonly utilized for physical activity. The largest percentage of adolescents’ vigorous physical activities occurred in the incidental open spaces (49.6%), followed by school playgrounds (40.1%). While most moderate physical activities occurred in neighborhood parks (38.6%) and school playgrounds (28.0%), the highest proportion of walking occurred in incidental open spaces (54.3%) and neighborhood parks (4.1%) |
| Oyeyemi et al 2014 [40] | Cross-sectional | Northeast/ Maiduguri | 1006 | 12-19 years | Physical Activity | Adolescent Activity Questionnaire for Adolescents and Young Adults (AQuAA) | Neighborhood Environment Features | Physical Activity Neighborhood Environment Scale (PANES) | Factors such as access to destinations (β = 0.18; CI = 0.67, 2.24), residential density (β = 0.10; CI = 0.01, 1.74), and the availability of infrastructure (β = 0.14; CI = 0.49, 2.68) were positively related to leisure-time MVPA and active transportation to school, but among boys only |

**Supplementary Table 2**. Sedentary behavior and social and physical environment factors

| Author/Year [References] | Study design | Region/City | Sample Size | Age | Outcome Variable | Measurement of outcome variable | Physical and social environment factors | Measurement of environment factors | Main findings with respect to environment factors and outcome variables |
| --- | --- | --- | --- | --- | --- | --- | --- | --- | --- |
| Ajayi 2021 [39] | Cross-sectional (mixed method) | Southwest/Osogbo | 1265 | 14-19 years | Sedentary behavior | Structured Questionnaire Survey of Sedentary Activities | Urban Open Spaces | Direct Observation; Satellite  imagery and  geographic information collected from Google Earth | Most of the participants (86.7%) engaged in sedentary activities. The most utilized open space typology for sedentary activities was the neighborhood park (92.1%) followed by pocket parks (88.2%), school playground (86.1%), and incidental open spaces (85.1%). |
| Odusoga & Sholeye, 2021 [41] | Cross-sectional | South West/ Ogun State | 330 | 15-19 years | Sedentary behavior | Validated 6-item Sedentary Behavior Questionnaire | Home and social environment features | Self-reported play objects at home, motorized transport at home, parental encouragement, and smoking and alcohol status | Over 90% of adolescents were sedentary. The high prevalence of sedentary behavior was due to screen-based behavior (79%), with phone-based behavior contributing the most (23.6%). Sedentary behavior was positively associated with ownership of motorized transport (p=0.038) and smoking (p<.001) |
| Otinwa and  Ademola, 2017 [42] | Cross-sectional | Southwest/ Lagos | 60 (30 males, 30 females) | 10-19 years | Screen time | Modified ICHPER-SD Children Screen Time Survey | Urban environment | City’s Urbanization Level determined by researchers | Screen time was high among urban living adolescents and was associated (p<0.05) with higher levels of Blood Pressure, BMI, waist-hip ratio, and lower physical fitness. |
| Ezezue et al., 2021 [43] | Cross-sectional | Southeast/ Enugu | 49 | 15-24 years | Sedentary time | Researcher-designed Questionnaire for physical activity and sedentary lifestyle | Home environment features | Self-reported spatial features/layouts of housing units and direct observations of building floor plans | Most (90%) of the time spent in home spaces was on sedentary activity (sitting). Only 7% engaged in light activity (standing), while 3% engaged in moderate activity. Those living in buildings with a traditional house layout were found to spend the least time on sedentary activity compared to those in dwelling units with a Western-style house layout pattern. |
| Anjana et al. 2024 [44] | Cross-sectional / multi country study | North East/ Gombe | 268 | 11-19 years | Sedentary behavior (screen time, transport-related sitting) | Validated Self-reported Screen Time Questionnaire: Accelerometer Measured Sedentary Time | Neighborhood and home environment features | Neighborhood Environment Walkability Scale for Youth (NEWS-Y); Rosenberg Brief Self-Report Measures of Home Electronic Equipment Environment | Significant positive associations between personal social media account and screen time were observed for Nigerian adolescents. Also, having electronic devices in the bedroom was significantly associated with higher screen time among Nigerian adolescents. The associations of transport-related sitting  time with pedestrian infrastructure and safety among  adolescents from Nigeria were positive, as were  those with neighborhood aesthetic |

**Supplementary Table 3**. Sleep and social and physical environment factors

| Author/Year [References] | Study design | Region/City | Sample Size | Age | Outcome Variable | Measurement of outcome variable | Physical and social environment factors | Measurement of environment factors | Main findings with respect to environment factors and outcome variables |
| --- | --- | --- | --- | --- | --- | --- | --- | --- | --- |
| Peter et al.,  2017 [45] | Cross-sectional | Northwest/ Kano | 353 | 10-19 years | Sleep pattern and sleep hygiene | BEARS Sleep  Screening Algorithm Questionnaire | Home electronic environment features | Validated Self-Reported Home Environment Sleep Hygiene Practice (e.g., use of bed time electronic device) | Awakenings during the night (34.6%) is the most common sleep-related problem reported, followed by excessive daytime sleepiness (21.0%). Although 62.9% of all the adolescents watched TV or played video games until 1 hr before going to bed, this was not statistically significantly associated with any sleep problems. |
| Olorunmoteni et al., 2018 [46] | Cross-sectional | Southwest/ Ile-Ife | 346 | 10-19 years | Sleep pattern | Adolescent Sleep  Habits Survey  Questionnaire (ASHSQ) | Home electronic and social environment features | Self-Reported Home Electronic Environment Component of ASHSQ; Self-Reported Information on Family Structure and Parents Social Class | Total sleep duration on weekends of 9h 08 min ± 113 min was higher than that of weekdays (7 h 15 min ± 86 min) (P < .001). Also, about half of the respondents (48.7 %) had access to a computer, and most of them (80.9%) used one or more electronic devices at bedtime. The proportion of adolescents with short sleep duration was 44.4% for weekdays and 5.5% for weekends. Lower social class (aOR = 2.706; 95% CI = 1.179- 6.212) and non‐use of computers at night (aOR=2.126; 95% CI=1.081-4.179) were predictors of adequate sleep duration on weekdays. In contrast, female sex (aOR = 1.733; 95% CI =1.069-2.811), younger age (aOR = 3.312; 95% CI = 1.316-8.333), and a polygamous family setting (aOR = 4.262 (95% CI = 1.825-9.956). were predictors of sufficient sleep on weekends. |
| Sanya et al.,  2015 [47] | Cross-sectional | Northcentral/ Ilorin | 1033 | 10-19 years | Sleep duration | Researcher Adapted Questionnaire on Sleep Habits | Home and social environments | Self-Reported Information on Home Electronic and Sleep Environments | About 13% of teenagers had insufficient nighttime sleep duration (<8 h), while the duration was adequate (>9 h) in 41% and borderline (8-9 h) in 44.3% (P < 0.05). Majority (76.2%) of teenagers co-share a bed with at least one person and 23.8% slept alone in bed. The leading reasons for going to bed were: Tiredness (31.1%), completion of house assignment (20.5%), and parental directive (12.4%). 10% of teenagers do make regular phone calls at night and 5.5% surf the internet and use computers at night. Regular habits of daytime sleepiness were reported by 8.2% of teenagers. Teenagers' mean sleep duration during school days was 9.33 ± 2.29 h compared to 10.09 ± 1.32 h at weekend (P < 0.05). |
| Omotosho et al., 2022 [48] | Cross-sectional | Northcentral/ Ilorin | 512 | 10-19 years | Sleep disorders | Pittsburgh Sleep Quality Index Questionnaire | Social environment | Self-Reported Information on Family Structure, and School Setting | Three out of every five adolescents were poor sleepers (PSQI global score > 5). Only a few adolescents (19%) had optimal sleep (≥ 9 hours), and more than half had inadequate sleep (< 7 hours). Poor sleep was associated with male gender, being the first born and residence at home. Logistic regression analysis showed that living at home, as against hostel living, was predictive of poor sleep. |
| Balogun et al. 2017 [49] | Cross-sectional | Southwest/ Ibadan | 450 | 10-19 years | Sleep patterns, problems, and quality | Pittsburgh Sleep Quality Index Questionnaire | Social environments | Self- Reported Information on Family Structure, Parents’ Social Class, and School Setting | The adolescents rated their sleep as very good (80.4%), fairly good (16.4%), fairly bad (0.9%), and very bad (2.2%). A higher proportion of adolescents spent a short time in bed before sleep, ≤15 min among those who had good sleep quality (81.4%) compared with those who had poor sleep quality (65.2%) (p < 0.001). The odds of having poor sleep quality were significantly higher in the subgroup who had ≤8.5 hours of sleep than those who had 8.5 hours of sleep or more (p < 0.001). School types and parents’ SES were not associated with sleep quality. |
| Olorunmoteni et al., 2023 [50] | Cross-sectional | Southwest/ Ile-Ife | 448 | 10-19 years | Sleep quality | Pittsburgh Sleep Quality Index Questionnaire | Social environments | Self- Reported Information on Family Structure, Parents’ Social Class, and School Setting | The majority of adolescents (85.0%) had poor sleep quality. More than half of adolescents (55.1%) had insufficient sleep during weekdays while only 34.8% had insufficient sleep during weekends. The school closing time and school type showed a statistically significant association with sleep quality (p= 0.039 and 0.005 respectively). The odds of having poor sleep quality increased by two-fold among adolescents in private schools compared with those in public schools (aOR=1.97, 95%CI=1.069 - 3.627). |
| Maduabuchi  et al. 2014, [51] | Cross-sectional | South East/ Enugu | 443 | 10-19 years | Sleep pattern | Questionnaire (Epworth Daytime Sleepiness Scale and Pittsburgh Sleep Quality Index) | Social environment | Self-Reported Information on Social Class | Twenty-six (5.9%) adolescents reported difficulty falling asleep. The mean duration of night sleep of the adolescents during weekday was 7.84 (1.9) hours and 8.65 (2.07) hours during the weekend. 22.8% adolescents had abnormal sleep onset latency (< 5 minutes and > 30 minutes). The social class and gender of the adolescents did not influence the sleep onset latency (χ2 = 32.89, p= 0.57). . |

**Supplementary Table 4**. Body weight status and social and physical environment factors

| Author/Year [References] | Study design | Region/City | Sample Size | Age | Outcome Variable | Measurement of outcome variable | Physical and social environment factors | Measurement of environment factors | Main findings with respect to environment factors and outcome variables |
| --- | --- | --- | --- | --- | --- | --- | --- | --- | --- |
| Ejike et al. 2008 [52] | Cross-sectional | Northcentral/ Lokoja, Ajaokuta, Ochaja | 1088 | 10-20 years | BMI | Objective Height and Weight measurements with calibrated scales research equipment | Urbanization | Town’s urbanization (urban vs non-urban areas) classification determined by researchers | Adolescents in the urban areas had higher BMI (20.74 ± 3.27 kg/m2 for males and 21.35 ± 3.37 kg/m2 for females) than those in the non-urban areas (20.33 ± 3.11 kg/m2 for males and 20.60 ± 2.97 kg/m2 for females), though the difference was significant (p < 0.05) only in the females |
| Olumakaiye. 2008 [53] | Cross-sectional | South West/  Osun State (25 urban areas, 7 rural areas) | 401 (182 boys,  219  girls) | 10-19 years | BMI | Objective Height and Weight measurements with calibrated scales research equipment by trained researchers | Urbanization | State Level Classification of Urban and Rural Districts | The prevalence of overweight among adolescents was 3.2%. Overweight prevalence was higher among those in urban (4.1%) than those in rural (1.5%) areas, and among girls (5.0%) than boys (1.1%). The prevalence of obesity was 0.5%. Only urban girls accounted for the prevalence of obesity. |
| Omigbodun  et al. 2010  [54] | Cross-sectional | South West/ Ibadan | 1799 (924 boys,  875  girls) | 10-19 years  (mean  15.0±2.3 years) | BMI | Objective Height and Weight measurement with calibrated scales research equipment by trained researchers | Urbanization, and Social environment | State Level Classification of Urban and Rural Districts; Self-Reported School Setting (public vs private schools) | 2.3% of adolescents were overweight. Girls were more likely to be overweight (AOR 3.5; 95% CI 1.7–7.3) while students in urban private schools had higher odds of being overweight than those in rural public schools (AOR 5.6; 95% CI 1.6–20.1). Adolescents in rural public schools were two times more likely to be underweight than those in private schools (AOR 2.3; 95% CI 1.3–3.9). |
| Ben-Bassey  et al. 2007  [55] | Cross-sectional | South West/  Lagos | 1504 (814  girls,  690 boys) | 10-19 years | BMI | Objective Height and Weight measurement with calibrated scales research equipment | Urbanization, and Social environment | City Level Classification of Urbanization (urban vs rural areas); Self-Reported Information on Parents’ Social Class | The prevalence of overweight and obesity is higher in urban areas (3.7% and 0.4%, respectively) than in rural areas (3.0% and 0.0%, respectively). No significant difference in BMI was found by gender and social class in the two areas. Altogether, the prevalence of overweight and obesity was 1.9% and 0.1%, respectively, for the boys and 4.7% and 0.25%, respectively, for the girls. |
| Adegoke et al. 2009 [56] | Cross-sectional | South West/  Ile-Ife | 720 | 6-18 years | BMI | Objective Height and Weight measurement with calibrated scales research equipment | Social environment | Self-Reported Information on Parents’ Social Class | The prevalence of overweight (2.8%) and obesity (0.3%) was low. Social class was associated with overweight, with a higher proportion of overweight students in the higher social classes when compared with the lower social classes (p=0.03). |
| Ojofeitimi et al. 2011 [57] | Cross-sectional | South West/  Osun State  (Olorunda LGA) | 520 (257  girls,  263 boys) | 10-19 years | BMI | Objective Height and Weight measurement with calibrated scales research equipment by trained researchers | Social environment | Self-Reported Information on Family Structure, Parents’ Socioeconomic Status (education), and School Setting (private vs public schools) | Overweight/ obesity had a significant association with the school type (X^2^=10.85; P=0.013). The majority of the girls from private schools were underweight (52.0%), 10 (4.0%) were overweight and 3 (1.2%) were obese. For public schools, the majority (55.4%) fell within the normal group, 6 (2.3%) were overweight and none was obese. |
| Ene-Obong  et al. 2012  [58] | Cross-sectional | South East, South-South, and South West (Aba,  Nsukka, Port  Harcourt,  Lagos) | 1599 (803  girls,  796 boys) | 5-18 years | BMI | Objective Height and Weight measurement with calibrated scales research equipment by trained researchers | Urbanization, and Social environment | City Level Classification of Urbanization; Parents’ Socioeconomic Status (income) | The prevalence rates of overweight and obesity were 11.4% and 2.8% respectively, and varied significantly (p = .001) according to location and socioeconomic status. The prevalence rate was highest in Lagos (Overweight = 23.6%,  Obesity = 10.2%), followed by Nsukka (Obesity = 12.3%, overweigh = 2.3%), and Port Harcourt (Obesity = 9.9%, overweight= 0.4%), and lowest in Aba (Obesity = 2.6%, overweight= 0.9%). The rates of overweight and obesity were highest in those with income >N300,000 (25.8%), followed by those with income between N100,000 and N300,000 (15.4%) and lowest among those with income of <N100,000 (7.1%) |

**Supplementary Table 5**. Wellbeing and social and physical environment factors

| Author/Year [References] | Study design | Region/City | Sample Size | Age | Outcome Variable | Measurement of outcome variable | Physical and social environment factors | Measurement of environment factors | Main findings with respect to environment factors and outcome variables |
| --- | --- | --- | --- | --- | --- | --- | --- | --- | --- |
| Modupe 2021 [59] | Cross-sectional | South West/ Ibadan, | 270 | 10-20 years | Social  Well-being | Validated Social Well-being Scale from the “School Location, Type, Juvenile Delinquent Behaviour and Social Well-being Questionnaire (SLTSWQ) | Social environment | Validated School Type Scale (STS) and School Location Scale (SLS) from the “School Location, Type, Juvenile Delinquent Behaviour and Social Well-being Questionnaire (SLTSWQ) | There was a significant relationship between school type and the social well-being of delinquent adolescents (r = 0.144, p=0.018). Also, there was a significant relationship between school location and the well-being of delinquent adolescents (r = -0.268, p=0.000). There was a common and relative effect of environmental factors (school type and school location) on the social well-being of delinquent adolescents: school type (β = .143, p<.05) and school location (β = -.239, p<.05). |
| Cheng et al. 2014 [60] | Cross-sectional / multi country study | South West/ Ibadan | 449 | 15-17 years | Hope, Depression, Posttraumatic stress | Validated Scales (Hope Scale; Center for Epidemiological Studies  Depression Scale; Post-Traumatic Stress Disorder (PTSD) Checklist) | Social environment | Researcher Designed and Validated Survey Assessing Self-Reported Social Support from Family, Peers, and Perceived Connection to Neighborhood | Greater feeling of connection with the neighborhood environment (β= 0.09, 95%CI=0.01, 0.17 in males), greater social support from caring female adults in the home (β= 0.08, 95%CI=0.06, 0.10 for females) were positively associated with higher level of hope among adolescents in Ibadan. |
| Nnubia &  Emmanuel  2023 [61] | Cross-sectional | South East/  Udenu LGA,  Enugu | 836 | 10-18 years | Anxiety, Depression | The 25-item Revised Child Anxiety and Depression Scale (RCADS 25) | Social environments | Pre-validated structured Questionnaire Assessing Family Structure, Emotional Connection with Parents, Parents’ Socioeconomic Status, and School Setting | Results showed a high (35.4%) prevalence of general anxiety and depression among adolescents. Males had a higher prevalence of anxiety (32.1%), depression (31.5%), and general anxiety and depression (44.8%) compared to females who had 20.9% symptomatic depression, 21.9% anxiety, and 29.2% general anxiety and depression. Among boys, general anxiety and depression was significantly associated with being in the lower age of 10 to 14 (r= -0.119), living with guardians (r=0.173), not having a close emotional connection with parents/ guardians (r=0.143, and coming from a low-income family (r= -0.169). Among girls, general anxiety and depression in girls were significantly associated with being in the older age range (r=0.167), being in senior secondary classes (r=0.132), and paradoxically, with having a close emotional connection with parents/ guardians (r=-0.110). |
| Alhassan et al 2024 [62] | Cross-sectional | North West/  Jigawa, Katsina,  Kaduna, Kano, Kebbi, Sokoto,  and Zamfara  States | 384 | High school adolescent students | Psychological well-being | Adapted and Validated Psychological Well-being Scale (PWS) Questionnaire | Social environment | Adapted and Validated Peer Pressure Scale (PPS) Questionnaire | There was a significant positive correlation (r= 0.658, p= 0.000) between peer pressure and poor psychological well-being of Senior Secondary School adolescents in North-west Nigeria. |
| Asukuti et al. 2023 [63] | Cross-sectional | North Central/ Ilorin | 400 | 10-20 years | Depression | Researcher Designed and Validated ‘Prevalence and Coping Strategies for Depression among In-school Adolescent Questionnaire (PCSDIAQ)’ | Social environment | Coping Strategy Scale of PCSDIAQ for Assessing Information on Sociobehavioral Coping Strategies and Social Interaction | Many in-school adolescents experienced depression symptoms, including fatigue (59.0%), a preference for solitude (50.3%), a lack of interest in or enjoyment from activities (48.8%), and anger for a few hours each day (more than 30%). For coping strategies, 42.3% always play games to distract themselves from problems. While 44.5% of avoided talking to people about their feelings for a short period, others criticized themselves (28.8%), isolated themselves (24.5%), avoided social situations until they felt better about themselves (15.8%), constantly blamed others (19.0%), and ignore the problems (10.0%). |
